# Estimating the potential of beekeeping to alleviate household poverty in rural Uganda

**DOI:** 10.1371/journal.pone.0214113

**Published:** 2019-03-27

**Authors:** Deborah Ruth Amulen, Marijke D’Haese, Eline D'Haene, James Okwee Acai, Jacob Godfrey Agea, Guy Smagghe, Paul Cross

**Affiliations:** 1 Department of Livestock Industrial Resources, Makerere University, Kampala, Uganda; 2 Department of Plants and Crops, Ghent University, Ghent, Belgium; 3 Department of Agricultural Economics, Ghent University, Ghent, Belgium; 4 Department of Extension and Innovation Studies, Makerere University, Kampala, Uganda; 5 School of Environment, Natural Resources and Geography, Bangor University UK, Gwynedd, United Kingdom; Institut Sophia Agrobiotech, FRANCE

## Abstract

Robust evidence underpinning the role of beekeeping in poverty alleviation is currently lacking. This study estimated the production potential for beekeepers in Northern Uganda by quantifying current production assets (equipment and knowledge) and impact on rural income streams range of proposed interventions. Intervention scenarios evaluated the economic benefits to be derived from different hive types combined with year-round provision of a nectar source (*Calliandra calothyrsus*) planted at varying density. Findings show that the type and number of beehive combinations used influenced the amount of revenue streams generated by the beekeepers. Addition of 20 log hives increased incomes 10 times, 20 KTBs increased revenues 16 times and Langstroth 18 times. Adding *Calliandra* trees as a forage source to the baseline scenario yielded revenues up to 17.6 times higher than the baseline. Implying that good management plus the introduction of a reliable nectar source, to off-set dry season challenges (absconding), could improve beekeeping productivity in Northern Uganda. Further research is required to validate *in situ* the impact of modelled scenarios on both honey yield and other ecosystem service benefits.

## Introduction

Beekeeping is thought to be a crucial component of livelihood diversification in sub-Sahara Africa, as it can supplement household incomes, food and medicine [1]. The relatively low start-up costs, labour requirements and minimum land ownership, render beekeeping an attractive economic pathway out of poverty for the rural poor, particularly women and young people [2]. Additionally, bees provide an important ecosystem service via pollination, directly contributing to enhanced food security, and increasing yields in ~75% of global crops [3].

East Africa has attracted substantial investment for beekeeping through donations and government interventions. Consequently, four East-African countries (Kenya, Tanzania, Uganda and Ethiopia) are licensed to export honey to the European Union [4]. In these countries, domestic demand exceeds available supplies of honey and hence unit prices per kg are elevated [5,6]. For example, in Uganda and Kenya the average price per kg honey is estimated at $6 US [7]. Suggesting that if beekeepers increased their honey production additional revenues would create positive change within poor households that live on less than $1.90 US a day. However, national and regional honey production remains constantly far below the potential supply levels [8]. All four nations are producing below their estimated production potential, i.e. Tanzania exploits 13.8% of 19,000 tonnes [9], Kenya 14.6% of the 100,000 tonnes [5] and Ethiopia 10.5% of the 500,000 tonnes [10]. Hence there is a need to develop suitable interventions that beekeepers can adopt to help meet the production capacity.

Ugandan beekeepers harvest just 1% of the estimated production potential of 500,000 tonnes [11]. Low production is attributed to several factors including weak policies, investment and knowledge exchange between stakeholders [6,12]. The greatest potential to increase beekeeping yields resides in northern Uganda where 60% of households own beehives [13]. Beekeepers in the region are constrained by forage seasonality, limited access to equipment, and training [2,14,15], accentuated by extended droughts and bush fires [16,17]. The benefits of planting forage crops to increase hive yields and colony survival are known [18]. Honeybees require diverse and constant supplies of protein (pollen) and carbohydrates (nectar) for optimal honeybee colony development, especially in the dry seasons where alternative forage sources are limited [19].

The success of a beekeeping activity in rural communities is contingent upon its profitability within the farmstead. This study modelled potential yield changes for three hive types, coupled with a dry season carbohydrate source to sustain colonies during periods of forage scarcity. Provision of a year-round bee forage plant was hypothesized to reduce absconding and increase colony survival leading to increased yields [20].

## Materials and methods

### Study area and data collection

Ethical approval was obtained from the college of Veterinary Medicine, Animal Resources and Biosecurity (COVAB) Makerere University (No. SBLS ADR 2016). Household questionnaires were administered to adult participants who were eighteen years and above. Before responding to the questionnaires both written and verbal consent was obtained. Verbal consent was captured in voice recorders. The interviewers ensured that questionnaires were administered in local languages so that participants understood what they were accepting to engage in.

**Fig 1:**
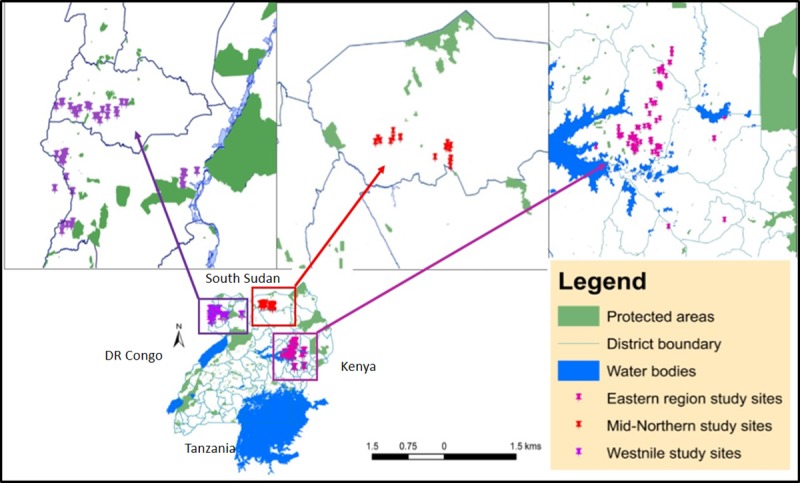
Map of Uganda showing the distribution of study sites Northern Uganda (West Nile, mid-northern and Eastern). The base map of protected areas was downloaded from world resource institute website [21].

The study was conducted in three agro-ecological zones of Northern Uganda (West Nile, mid-northern and Eastern) (Fig 1). Socio-demographic profiles of beekeepers, knowledge, equipment and product yields data was generated through a cross-sectional household survey and literature review captured the nectar production potential of *Calliandra*. Subsequently, the cost benefit analysis and Monte Carlo simulations.

### Household survey

A four-month cross-sectional household study was conducted between November 2014 and February 2015 in three agro-ecological zones of Northern Uganda. The zones were purposively selected based on mean annual honey yields [13]. The West Nile (84,320 kg), was classified as a relatively high producer, the Mid-Northern (27,500 kg) as moderate and Eastern (16,310 kg) as a low producer [13]. One hundred and sixty-six beekeepers (from a national list of 630 beekeepers registered by the Uganda National Apiculture Organization (TUNADO), from the three study zones [West Nile (n = 59), Eastern (n = 69) and Mid-Northern (n = 38)].

A semi-structured questionnaire (S1 Questionnaire) collected information regarding beekeeping knowledge, experience, equipment owned and quantity of honey produced annually. Forty-two variables (S1 Table) were used to characterise the social and demographic profiles of the beekeepers, their knowledge, equipment and hive-product yields. Hive product quantities and their economic value were calculated in order to estimate the current contribution of beekeeping to households. An adoption variable was included to represent the potential behavioural changes that beekeepers undergo when accepting new technologies such as adoption of modern hives or techniques [22]. Understanding such levels of behavioural change within a target community can potentially shape the size and extent of a required development intervention [22].

Any differences in socio-demographic variables were estimated by Pearson chi-square statistics estimated in SPSS 22 [23]. A Mann-Whitney U test was used to determine differences in the type and quantity of beekeeping equipment and yield across adopter categories [23]. Results from the household survey formed the baseline information for the cost-benefit analysis.

### Cost-benefit analysis of interventions

Cost-benefit analysis evaluated the economic effectiveness of each intervention (changing hive type, forage provision and knowledge) [24]. Predicted future incomes were modelled for a set temporal periods and reported as net present value (NPV) [24]. The investment costs, future costs and benefits were calculated [24]:

$$NPV=\sum_{t=1}^{n}\frac{B_{t-C_{t}}}{(1+r)t}+\frac{R_{n}}{(1+r)t}-A$$

Where B_t_ = benefit in year t, C_t_ = investment and recurrent cost in year t, r = discount rate, R_n_ = rest value of the investment in year n, and A = investment in year 0.

### Costs and benefits

Basic beekeeping entails the acquisition of beehives, smokers and protective suits and gloves [11]. In establishing an apiary, a beekeeper incurs an initial labour cost to clear vegetation that might hinder the movement of the beekeeper when inspecting the hives [2]. Once the hives have been installed, they require routine inspection for pest management and to monitor colony performance which incurs further labour costs [25]. Honey harvesting incurs several additional costs such as labour, honey storage equipment (airtight buckets) and a torch (as hives are generally inspected at dusk due to the bees’ defensive behaviour) [26]. For scenarios that included a forage crop, there was additional seedling purchase costs, maintenance and planting [27]. Benefits comprised honey, beeswax and occasional propolis harvesting.

### Interest rates, time and unit prices

The 10% discount rate included in calculations was based on lending rates from the Centenary Bank for the year 2014 [28]. This bank commonly offers loans for beehive purchases to farmers in Northern Uganda, particularly in the West Nile region [29]. A repayment period of 10 years was modelled and all monetized values were converted from local currency to US dollars (Ugandan shillings 2700 = 1 US dollar; mean exchange rate 2014). The unit prices for inputs and products were based on the prevailing market prices at the time of the study.

### Supplementary assumptions

For all scenarios, derived benefits commenced in the second year. After an initial investment, beekeepers were expected to achieve 30% of total potential production followed by an annual increment of 15% of the previous year’s honey yields from years three to six; remaining constant from years six to eight and decreasing by 15% in years nine and ten. These assumptions are based on regional beekeeping practices where hive colonisation is contingent upon swarms occupying empty hives [20]. The quantity of propolis produced was held to be constant over the period [12]. Bee hives were considered to have no resale value by the 10^th^ year due to natural depreciation.

### Six cost-benefit intervention scenarios

#### Scenario 1: The baseline (current state of beekeeping)

Costs and benefits under this scenario were based on estimates derived from the household survey. The average number of beehives managed per beekeeper was 22 (s.d. = 15.6) on 3.7 ha of land. Apiaries typically comprised traditional fixed comb hives (log hives (mean = 14, s.d. = 13)), followed by removable top bar hives (KTB and other related hives (mean = 7, s.d. = 8)) and one removable frame hive (Langstroth (mean = 4, s.d. = 5)). Other equipment included a smoker (mean = 1, s.d. 5.41), a bee suit including gloves, a pair of gumboots and an airtight bucket for honey storage. The mean unit costs of production equipment were as follows: log hives ($3.42 US, s.d = 2.01), KTB and other related beehives ($36.64 US, s.d. = 25.62), Langstroth and other related beehives ($43.24 US, s.d. = 33.62), smoker ($11.44 US, s.d. = 5.41), bee suit ($46.21 US, s.d. = 29.65), pair of gumboots ($6.24 US, s.d. = 1.54) and airtight bucket ($7.84 US, s.d. = 1.11). Three products were harvested, the mean yield of which was honey (13.42 kg/year, s.d. = 17.80), beeswax (3.51 kg/year, s.d. = 16.10) and propolis (0.19 kg/year, s.d. = 1.04). The unit price of honey was $2.61 US/kg (s.d. = 1.72), beeswax was $3.01 US/kg (s.d. = 1.13) and propolis was sold at $4 US/kg (s.d. = 2.66).

Whereas beekeeping is known as a less labour intensive activity compared to other agricultural activities like livestock keeping [1], routine inspections to control pests and monitor progress of honey yields are essential to good practice [1]. Therefore, other cost variables in this scenario included bee colony maintenance such as routine inspections, equipment repairs, torches and batteries. Maintenance costs were assumed to be incurred annually. Unit labour costs were based on the hourly rate equivalent paid to casual labour in the survey villages ($2 US/day) [27]. Inspection costs were allocated separately to harvesting costs, because experienced beekeepers were hired instead of casual labour to help with honey harvesting [11]. From the household survey, 57% of the beekeepers rarely inspected their colonies. Only 45% of beekeepers harvested honey twice a year. We assumed that beekeepers inspected their colonies and apiaries six times per year ($2 US/inspection). Frequent inspection of honeybees, e.g. every two weeks, is discouraged because of the bees’ high absconding rate if repeatedly disturbed [20]. Honey harvesting is considered the most dangerous and difficult of all the apiary tasks and is therefore relatively expensive. Honey harvesting is in most cases paid in-kind (honey) [1]. The two harvesting seasons in the region incur a cost of $2 US/day and require approximately five hours work to collect honey from 22 hives.

Honey is highly hygroscopic (absorbs moisture) and needs to be stored in airtight buckets to avoid fermentation [1]. It was assumed a beekeeper would own airtight buckets with a storage capacity of 20 kg per bucket. Beekeepers would need two strainer cloths to filter the honey, for every 400 kg of honey processed [1]. Gumboots were replaced every three years. Annual marketing costs were estimated to be 10% of total revenue [1]. Unit product prices were assumed constant for the years of intervention (Table 1).

**Table 1:** Mean cost for modelled scenarios*.

	Scenario 1 (base line)	Scenario 2 national average yield	Scenario 3d (20 log hives)	Scenario 4d (20 KTB hives)	Scenario 5d (20 Langstroth hives)	Scenario 6d (2000 *C*. *calothyrsus* trees)
***Hypothesised revenue***						
**Honey sales**	61	413	726	530	1000	486
**Beeswax sales**	18	48	84	612	115	56
**Propolis**	0.8	0.8	0.8	0.8	0.8	0.8
**Total benefits (Annual average)**	81	461	811	1143	1116	543
***Bee colony maintenance and product harvest***						
**Labour for routine inspection @ per man hour**	24	92	118	120	120	92
**Labour for product harvest processing and packaging**	14	68	117	140	159	68
**Lighting and batteries**	4	10	10	10	10	10
**Gumboots pairs**	1	1	1	0.6	0.6	0.6
**Marketing**	8	46	74	98.5	112	46.1
**Straining cloth**	0	0.4	1	0	2	0.4
***Calliandra additional costs***						
**Plant weeding and maintenance**						60
**Total variable costs (a)**	51	217	322	369	402	277
**Investment costs**						
**Log beehives**	48	47.88	116	48	47.88	48
**Kenya top bar hive**	256	256.48	256	989	256.48	256
**Langstroth**	43	43.24	43	43	908.04	43
**Smoker**	11	11.44	11	11	11.44	11
**Bee suits pairs**	46	46.21	46	46	46.21	46
**Airtight buckets**	8	82.71	259	177	200.31	93
***Beehive siting***						
**Site clearing**	4	4	12	16	16	4
**Labour for hanging of beehives**	4	4	12	16	16	4
***C*. *calothyrsus* costs**						
**Seedling purchase**						60
**Seedling planting**						200
**Total fixed costs (b)**	421	496	75	1345	1502	766

* Vales (USD) and calculated for a 10-year cash flow. For each suggested intervention, the optimal scenario (highest benefits) was chosen and mean annual cost for each input computed.

#### Scenario 2: Beekeepers increase honey yields to the national average level

Scenario 2 investigated the potential of beekeepers in the study region to increase their yields to the national average yield level per hive type. The projected honey yield from each hive was based on the following national mean yields for the three hive types: Langstroth: 15 kg/hive/year; KTB: 12 kg/hive/year; and the log hive: 8 kg/hive/year [13]. It was assumed that beekeepers would harvest and process pure wax for sale rather than the current practice whereby 71% of wax is sold in honey and comb products. Estimation of available pure wax was based on 10 kg of honey being sufficient to produce 1 kg of beeswax [25].

An increase in honey production requires a corresponding increase in the number of storage containers and filters. Inspection and honey harvesting costs for the beekeepers in this scenario increased to four times/month at $2 US per visit (as compared to six times a year in scenario 1). Marketing and harvesting-related labour costs were assumed to increase by 10% annually owing to the increased number of colonised hives (Table 1).

#### Scenarios 3, 4, 5: The effect on income of changing hive type and number

We modelled productivity changes of the three hive types mentioned in Scenario 1. Scenario 3a to 3d evaluated the economic potential of the traditional beehive (fixed comb) at four different hive numbers (5, 10, 15 and 20 hives). This was repeated for removable fixed comb hives (Langstroth and related beehives) in scenario 4; and removable top bar hives (KTB and related beehives) in scenario 5. Thereafter, their productivity was compared to determine the Net Present Value (NPV) (Table 1).

#### Scenario 6: The effect on honey yield of providing a nectar crop (bee forage)

Maintaining out of season forage access for bees is a significant challenge for Ugandan beekeepers [30]. Due to forage scarcity, high absconding and low hive colonisation rates are common place [20]. We modelled the effects of adding *C*. *calothyrsus*, a year-round nectar source in East Africa, to the forage matrix. The number of flowers produced per day from a *C*. *calothyrsus* tree have been recorded at between 1–34 with a nectar volume of 20–50 μl per flower per day [31,32]. The following three assumptions were made: an average nectar flow of 35 μl per flower at a sugar concentration of 20% with an average of 17 flowers per plant per day [19,33]. The calculated total mg of sugar per flower converted to honey was based on the exponential regression equation:- y = 0.00266 + (0.00937x) + (0.0000585x^2^), where x is the concentration of nectar as read from a refractometer and y is the mg of sugar per μl of nectar [34]. For *C*. *calothyrsus*, a total of 0.2162 mg of sugar per μl of nectar was estimated. A total of 0.046 kg of sugar was expected from a single established *Calliandra* plant per annum. *C*. *calothyrsus* requires approximately 2.25 m^2^ for each plant as recommended by [34].

Land availability determines the number of trees that can be planted: 0.1 ha for 500 trees, 0.2 ha for 1000 trees, 0.3 ha for 1500 trees and 0.5 ha for 2000 trees. We developed four scenarios for *C*. *calothyrsus* planting configurations and determined the subsequent NPV a beekeeper could expect to generate. It was assumed that beekeepers would plant *C*. *calothyrsus* between their gardens, which are normally leave uncultivated. Thus, if a beekeeper planted 1500 *C*. *calothyrsus* trees, the annual available nectar available to honey conversion would be 0.046 kg per plant per year x 1500 trees = 69 kg. As honeybees compete for nectar with other insects and bats [35], the available nectar to the bees was assumed to be reduced to 60% [35]. This reduces the potential extra yield of available honey to 41.4 kg. Unit costs per *C*. *calothyrsus* tree were estimated at $0.1 US while planting and weeding costs per seedling were $0.03 US per plant [27].

The underlying assumptions in this intervention are that there was sufficient pollen diversity and other nectar sources during the productive season and that *C*. *calothyrsus* trees mainly served in the maintenance of the colony during periods of scarcity. Because *C*. *calothyrsus* flowers after the first year [33] and nectar yields vary between individual plants, it was assumed that only 60% of total nectar would be obtained from the trees in years 1 and 2, followed by 80% in subsequent years when tree maturation completes.

### Monte Carlo modelling to simulate the range of NPV

We incorporated a probabilistic function to estimate the NPV due to fluctuations in input prices, variable costs and expected product yields [36]. Conventional cost-benefit analyses report deterministic NPVs, which assume static outputs, yet most outputs vary. Consequently, a stochastic Monte Carlo model was used to generate the NPV based on a random outputs model with a normal distribution function [36,37]. Cash flows for each scenario were calculated, as well as the minimum and maximum value of NPV (growth rate of the annual honey yields was assumed to be 15%). For each scenario, a minimum and maximum value of the NPV was generated before adding a distribution formula (triangular distribution) [= RAND x (Maximum value—minimum value) + minimum value] as proposed by [37]. A 10,000 iteration model generated the NPV for each scenario.

## Results

### Demographic profile of beekeepers

Approximately 74% of the beekeepers were aged 55 or younger. Most beekeepers were male (78%). Only 41% of the study population had attained any education mainly at primary level. Most beekeepers were late adopters (44%) having less than 3 years of experience in beekeeping, followed by early adopters (31%) which is a group that had 4 to 7 years, and finally innovators (25%) having more than 8 years of experience in beekeeping (Table 2). Most beekeepers (68%) on average owned 22 beehives (Table 3).

**Table 2:** Socio-demographic characteristics of beekeepers.

Socio demographic variable		Adopter categories based on years in beekeeping			Distribution of socio variable across adopter categories (χ^2^)
	All beekeepers (n = 166) percent	Late adopters, 1 to 3 yrs. (n = 72) percent	Early adopters, 4 to 7 yrs. (n = 52) percent	Innovators, >8 yrs. (n = 42) percent	
Age categories (years)					χ^2^ = 14.29**
17–35	31	59	22	19	
36–55	43	44	26	30	
56–70	16	28	50	22	
>70	10	18	46	36	
Gender					χ^2^ = 2.812
Female	22	56	22	22	
Male	78	40	34	26	
Education					χ^2^ = 13.14*
No-formal	59	52	25	23	
Primary	36	22	33	45	
Secondary	4	15	60	25	
Tertiary	0.6	0	100	0	
Agro-ecological zone					χ^2^ = 16.06**
Mid-Northern	23	71	16	13	
Eastern	42	33	39	28	
West Nile	35	38	31	31	
Scale of production					χ^2^ = 16.17**
small scale (<22beehives)	68	53	29	18	
Large scale (>beehives)	32	23	35	42	

Significant at

*10%

** at 5%

**Table 3:** Distribution of beekeeping equipment.

Equipment owned and their unit costs	Number of beekeepers (n)	Mean ± SD	Early adopters, >4 yrs, mean ± SE n = 93	Later adopters, <3 yrs, mean ± SE n = 73	Mann-Whitney U test- t statistic
Number of:					
All beehives types	163	22.00±15.60	28.00±2.77	16.04±1.58	4.23*
Log hives	153	14.49±12.94	19.00±1.63	10.00±1.34	4.23*
KTB hives	109	7.01±8.44	12.00±2.54	9.00±1.26	1.66.00
Langstroth hives	34	1.01±5.17	4.00±1.03	4.00±1.12	76.00
Pairs of gumboots	58	1.30±0.59	1.00±0.82	1.00±0.17	297.00
Bee suits	45	1.40±0.82	1.00±014	2.00±0.22	157.00
Smokers	41	1.50±0.48	1.00±0.70	1.00±0.26	136.00
Pairs of gloves	38	1.40±0.6			
Airtight buckets	32	2.10±1.84	2.00±0.38	2.00±0.27	132.00
Bee brushes	27	1.60±1.73			
Honey strainers	10	1.10±0.32	1.00±0.40	1.00	12.00
Hive tools	9	1.10±0.32			
Honey extractors	1	0	0.01±0.10	0	3.43*
Unit cost (USD) of equipment					
Log beehives		3.42±2.01			
KTB hives		36.64±27.62			
Langstroth hives		43.24±33.62			
Gumboots		6.26±1.54			
Bee suits		46.21±29.65			
Smokers		11.44±5.41			
Pairs of gloves		5.00±0.46			
Airtight buckets		7.84±1.11			
Honey strainers		24.07±1.5			
Hive tools		3.33±3.53			

Significant

* at 10%

** at 5% and

*** at 1% level (significance indicates mean difference between early and late adopters).

The unit prices are compiled for those beekeepers that purchased the beekeeping equipment themselves rather than receiving them cost free from donors.

### Beekeepers’ knowledge and group membership

**Fig 2:**
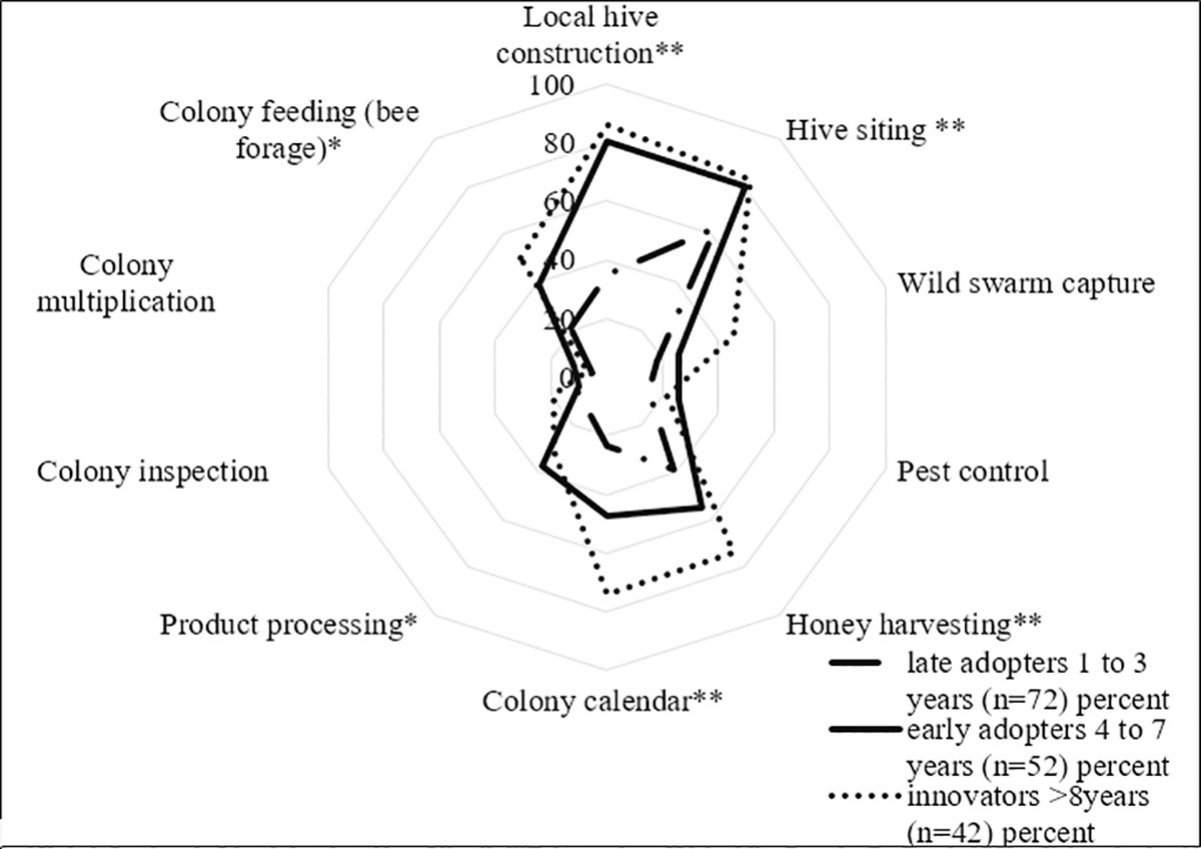
Beekeepers’ knowledge across adopter categories. Each line type represents an adopter category. Dotted line represents the level of beekeeping knowledge. The outer ring of the dotted web indicates the highest level of knowledge, whilst the most inner ring indicates the lowest level of knowledge. Pearson’s chi-square test of distribution. Significance level = *10%, ** at 5% and *** at 1% indicate significant differences of knowledge types held.

Beekeepers held knowledge about local hive construction, honey harvesting, hive-siting and bee forage requirements. Colony multiplication, inspection and pest control was the least held knowledge (Fig 2). Most beekeepers were members of a beekeepers’ group within the local community (n = 149), but fewer were members of a savings group (n = 77) or a marketing group (n = 16).

### Beekeeping physical assets (land and equipment)

Beekeepers owned an average of 3.72 hectares per household and allocated 2.21 hectares to crop production, 0.93 hectares to livestock and the remainder for homestead use. Most beekeepers owned log hives (94%) and/or Kenyan Top Bar and related hives (67%). Ownership of log hives was highest among beekeepers who held more knowledge about local hive production. Only 28% of beekeepers had protective suits, whilst 36% owned a pair of gumboots and 27% had a bee-smoker in their apiary. Only 19% of beekeepers owned equipment such as airtight buckets, honey strainers, honey presses and extractors (Table 3)

### Unit prices of harvested products

Honey, beeswax and propolis were the most commonly harvested products (Table 4) and their contribution to annual household revenues was 7%,

**Table 4:** Harvested products and unit prices.

Variable	Annual yield per beekeeper, mean ± S.D. (n = 163)
Products (annual yields, kg)	
Honey	13.42±17.80
Beeswax	3.51±16.10
Propolis	0.19±1.04
Current income benefit (annual income, USD)	
Total household income	615.48±947.16
Honey	32.10±30.43
Beeswax	10.33±43.74
Propolis	0.58±0.57.40
Total beekeeping income	43.01±79.24
Proportion of beekeeping income	0.69
Unit prices of product (USD/kg)	
Honey	2.61±01.72
Beeswax	3.01±1.13
Propolis	4.00±2.66

### Income generating interventions for beekeepers

The cash flow analyses (Table 5) revealed that changing the hive type from a traditional (log hive) to a frame hive (Langstroth) and/or top bar (KTB and similar hives) was the most profitable intervention over a 10-year investment. Except for the baseline scenario, all other scenarios demonstrated an increase in NPV, suggesting that if beekeepers adopted any of the modelled scenarios they could potentially increase their revenues (Table 5). Beekeepers could have increased profitability by augmenting the number of traditional hives without adopting any new hive systems, as three traditional hives had the same productive capacity as one frame hive.

**Table 5:** Income generating interventions for beekeepers*.

Suggested intervention	Year 0	Year 1	Year 2	Year 3	Year 4	Year 5	Year 6	Year 7	Year 8	Year 9	Year 10
Hypothesized honey yield increase	0	30%	45%	60%	75%	90%	90%	90%	90%	90%	90%
Baseline	-459	15	17	19	16	9	7	5	4	3	13
National average yield	-608	1	68	123	163	85	65	49	38	29	126
5 log hives	-805	19	98	162	209	106	81	62	47	36	158
10 log hives	-873	28	119	191	248	125	96	73	56	43	186
15 log hives	-948	37	140	224	287	144	110	84	64	49	214
20 log hives	-906	47	162	255	327	163	124	95	72	55	242
5 KTB hives	-1005	28	112	181	232	117	89	68	52	40	174
10 KTB hives	-1042	46	148	231	294	147	112	85	65	50	218
15 KTB hives	-1281	64	184	281	356	176	135	103	78	60	262
20 KTB hives	-1499	139	298	427	525	255	195	149	114	87	379
5 Langstroth hives	-868	34	123	195	249	125	95	73	56	42	186
10 Langstroth hives	-1125	59	170	260	328	163	124	95	72	55	242
15 Langstroth hives	-1399	84	217	324	407	200	153	117	89	68	297
20 Langstroth hives	-1656	109	263	389	486	238	181	138	106	81	353
500 *C*. *calothyrsus* trees	-683	-2	70	130	173	90	69	52	40	22	58
1000 *C*. *calothyrsus* trees	-748	-5	72	136	183	95	73	55	42	23	61
1500 *C*. *calothyrsus* trees	-813	-8	74	142	192	100	77	58	45	24	65
2000 *C*. *calothyrsus* trees	-878	-11	76	148	202	105	80	61	47	26	60

* All interventions are modelled at a discount rate of 10% over 10 years (USD)

The addition of *C*. *calothyrsus* maximised the NPV when planted at a density of 1,500 per ha. Beyond this level, marginal rates of return begin to diminish (Table 5). The least beneficial scenarios were the ‘business as usual’ baseline (scenario 1); beekeepers achieving national average yields per hive (scenario 2), and the addition of 1000 *C*. *calothyrsus* shrubs (scenario 3b) (Table 6).

**Table 6:** Ranking of the interventions based on NPV*.

Suggested intervention	Initial investment cost	Rank based on mean NPV	Mean NPV (USD)	SE
20 Langstroth hives	1,656	1	960	17
20 KTB hives	1,499	2	855	16
15 Langstroth hives	1,399	3	789	15
15 KTB hives	1,281	4	739	14
10 Langstroth hives	1,125	5	640	12
10 KTB hives	1,042	6	595	11
5 KTB hives	1,005	7	564	11
15 log hives	948	8	559	10
20 log hives	906	9	530	9
5 Langstroth hives	868	12	508	9
10 log hives	873	11	503	9
5log hives	805	14	476	9
1500 *C*. *calothyrsus* trees	813	13	378	7
2000 *C*. *calothyrsus* trees	878	10	376	7
500 *C*. *calothyrsus* trees	683	16	370	7
1000 *C*. *calothyrsus* trees	748	15	361	7
National average yield	608	17	357	6
Baseline	459	18	-196	4

*the ranking is based on the mean NPV using Monte Carlo analysis (95% confidence interval)

### Effect of increasing honey yields to the national average

**Fig 3:**
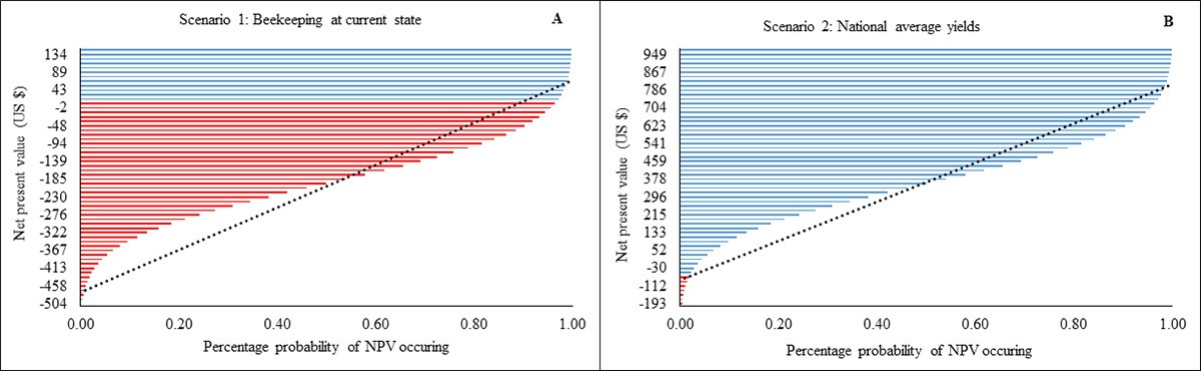
Probability (%) changes in Net Present Value (NPV) for changing current situation (Scenario 1) to obtain national average yields (Scenario 2). Red lines indicate loss and blue lines profit. Dotted black line is a linear forecast. NPVs to the right of the line indicate optimal profit/loss attained for each an intervention level.

Outputs from scenario 1 suggest that most beekeeping enterprises adopting this approach are running at a loss, as almost all the NPVs on the right-hand side of the linear forecast line are negative (lowest -183, highest $43 US indicated by the points where the line cuts the NPV) (Fig 3). Beekeepers lose up to $504 US and can gain up to $134 US, with 80% of beekeepers predicted to be loss-making. For scenario 2, most NPVs were positive (indicating profits) and there was a 50% probability that beekeepers’ NPV values (values on the right-hand side of the forecast line) would fall between a minimum profit of $378 US and maximum of $756 US. The maximum possible loss is reduced to $193 US.

### Changing type and number of hive scenarios

**Fig 4:**
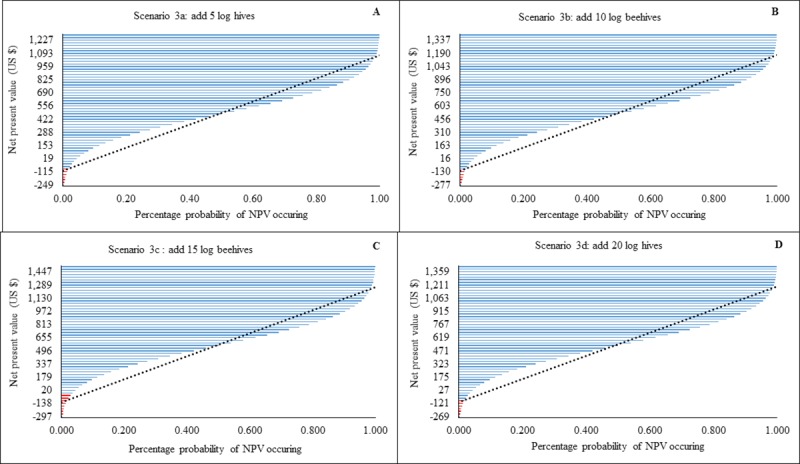
Modelled probability (%) changes in Net Present Value (NPV) for changing log hive numbers (scenarios 4a-d). Income changes following addition of log hives. Red lines indicate loss and blue lines profit. Dotted black line is a linear forecast. NPVs to the right of the line indicate optimal profit/loss attained for each an intervention level.

In scenarios 3a-d, the probability of beekeepers losing money was less than 20%. By adding 5 log hives, 50% of the NPVs would range between $422 to $959 US (estimates to the right of the forecast line), with an estimated maximum possible loss of $249 US (Fig 4A). With the addition of 10 log hives, there was a 50% chance that NPVs would occur between $456 and $1043 US with a possible maximum loss of $277 US (Fig 4B). By adding 15 log hives, 50% of the NPVs ranged between $496 and $1130 US with a predicted maximum loss of $297 US (Fig 4C). With the addition of 20 log hives, 50% of the NPVs ranged between $471 to $1063 US and the maximum predicted loss estimated to be $269 US (Fig 4D).

**Fig 5:**
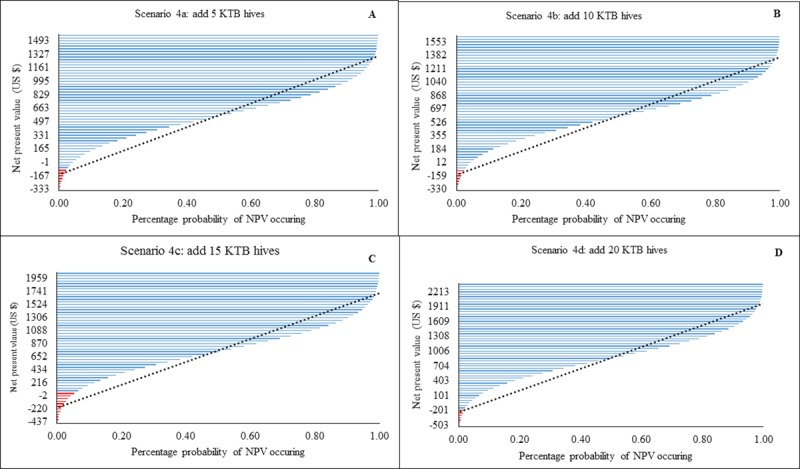
Modelled probability (%) changes in Net Present Value (NPV) for KTB hive numbers (scenarios 4a-d). Income changes following addition of log hives. Red lines indicate loss and blue lines profit. Dotted black line is a linear forecast. NPVs to the right of the line indicate optimal profit/loss attained for each an intervention level.

All versions of scenario 4 were profitable with a substantially reduced probability of loss compared to scenario 1. By adding 5 KTB hives, there was a 50% chance that generated NPVs would range between $497 to $1161 US, with a probable maximum loss of $333 US (Fig 5A). With the addition of 10 KTB hives, 50% of the modelled NPVs would range from $526 to $1211 US with a possible maximum loss of $330 US (Fig 5B). By adding 15 KTB hives, 50% of the NPVs would range between $652 to $1524 US with a predicted maximum loss of $437 US (Fig 5C). With the addition of 20 KTB hives, 50% of the NPVs would range between $503 to $1760 US and the maximum predicted loss was expected to be $503 US (Fig 5D).

**Fig 6:**
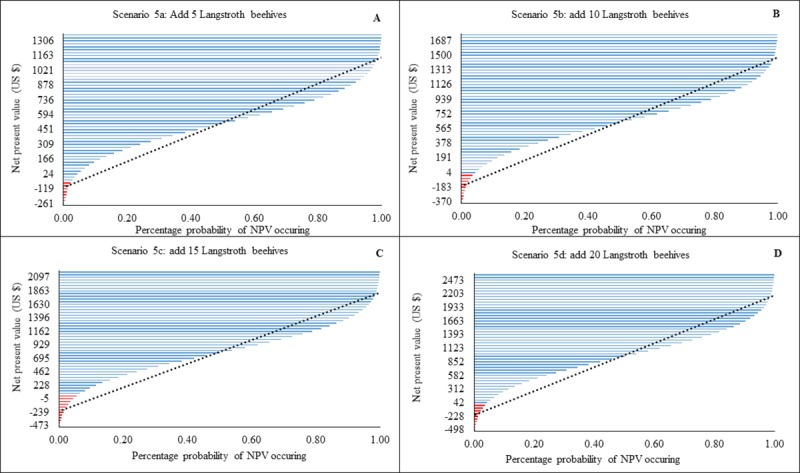
Modelled probability (%) changes in Net Present Value (NPV) for Langstroth numbers (scenarios 5a-d). Income changes following addition of frame hives (Langstroth and related hives). Red lines indicate loss and blue lines profit. Dotted black line is a linear forecast. NPVs to the right of the line indicate optimal profit/loss attained for each an intervention level.

All versions of scenario 5 had a higher profitability ratio with a substantially reduced likelihood of loss compared to scenario 1. With the addition of 5 Langstroth hives, there was a 50% probability that NPVs would range between $451 to $1021 US, with a maximum possible loss of $261 US (Fig 6A). By adding 10 Langstroth hives, there was a 50% likelihood that beekeepers NPVs would range between $565 to $1313 US and the maximum possible loss of $370 US (Fig 6B). Adding 15 Langstroth hives suggested that 50% of the NPVs would range between $695 to $1630 US with a possible maximum loss of $473 US (Fig 6C). By adding 20 Langstroth hives, 50% of the NPVs would range between $852 to $1933 US and the maximum predicted loss was $498 US (Fig 6D).

### The effect on income of providing a nectar crop (bee forage)

**Fig 7:**
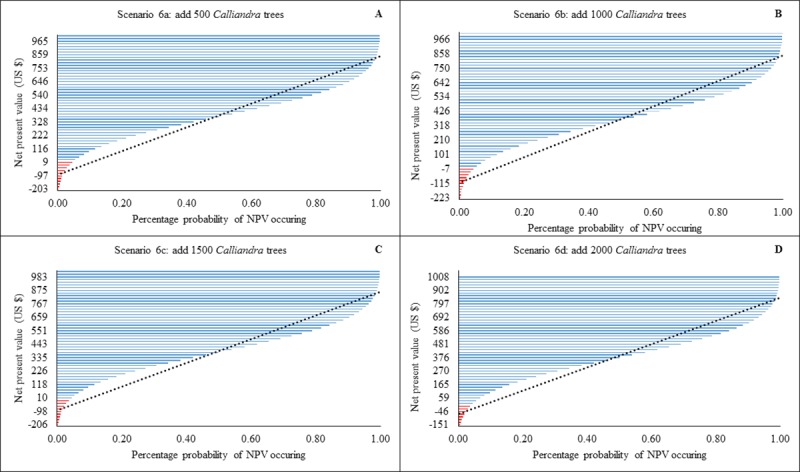
Modelled probability (%) changes in Net Present Value (NPV) for scenarios 6a-d of providing a nectar crop (*C*. *calothyrsus*). Red lines indicate loss and blue lines profit. Dotted black line is a linear forecast. NPVs to the right of the line indicate optimal profit/loss attained for each an intervention level.

All versions of scenario 6 were profitable with substantially reduced probability of loss compared to scenario 1. By adding 500 *C*. *calothyrsus* trees, there was a 50% chance that generated NPVs would range between $328 to $753 US, with a probable maximum loss of $203 US (Fig 7A). With the addition of 1000 *C*. *calothyrsus* trees, there was a 50% chance that beekeepers’ NPVs would range between $426 to $750 US and their maximum loss would be $233 US (Fig 7B). By adding 1500 *C*. *calothyrsus* trees, 50% of the NPVs would range between $335 to $767 US with a predicted maximum loss of $206 US (Fig 7C). By adding 2000 *C*. *calothyrsus* trees, 50% of the NPVs will range between $376 to $797 US and the maximum predicted loss is about $151 US (Fig 7D).

## Discussion

This study quantified the potential for beekeepers in Northern Uganda to enhance their economic resilience through beekeeping. To maximize income generation, beekeepers may need to adjust current management practices such as improved pest control, timely harvest, the number and type of hives as well as incorporating a year-round forage supply.

The models demonstrate that improved management practices (pest control and timely harvesting) and increased current incomes streams seven times (Fig 3). An outcome not surprising since pests and diseases have been cited as a major limiting factor to honey production [2,25,38]. Suggesting the need for the beekeepers to improve their pest and disease control strategies to realise the benefits of beekeeping.

The type and number of beehive combinations used influenced the amount of revenue streams generated by the beekeepers. For example, addition of 20 log hives increased incomes 10 times (Fig 4), while addition of 20 KTBs increased revenues 16 times (Fig 5) and Langstroth 18 times (Fig 6).Whilst frame and top bar hives (e.g. KTB, Langstroth) increase yields, we suggest that in areas where modern hive construction is challenging due to a lack of appropriate skills, beekeepers should augment the number of traditional hives at a ratio of three traditional to one modern. Traditional hives in sufficient number have the potential to augment current incomes without the unnecessary expenditure of purchasing frame hives such as the Langstroth. This is contrary to the notion promoted by some development agencies that significantly increased honey production is only possible through top bar and frame hive adoption [39,40]. Whilst traditional hive inspection is challenging to the beekeeper [14], their adoption should be encouraged for novice beekeepers (‘late adopters’ i.e. beekeepers with less than three years’ experience) who often lack sufficient financial capital and the requisite skills to acquire and manage fixed-frame hives.

The adoption of traditional hives has important cost-saving and pedagogical implications for both beekeepers and development agencies who tend to distribute frame hives without sufficient consideration or provision of the associated training requirements [41]. Development programmes tend to target regions where beekeeping is dominated by the poorest households [12]. Such impoverished households and communities tend to have reduced access to those supporting services needed for frame hive beekeeping (e.g. hive construction and maintenance). Development agencies may need to consider targeting the training of local traditional hive makers to increase the supply of hives in order to reduce the costs of construction. For instance, experienced beekeepers, with local knowledge of hive construction, are pivotal to both post-development programme training and hive construction and in this study tended to own more hives compared to other adopter categories (Fig 2).

The majority of surveyed beekeepers in the region had limited knowledge of both pest control and colony multiplication (see Fig 2). As such, most frame hives in the study area were either abandoned or ineffectively managed, in part due to a lack of protective equipment, but also owing to a lack of the requisite skill set required to effectively manage colonies [12,30,42].

For beekeepers whose skills are more advanced (early adopters and innovators), it may be appropriate to encourage the combined use of modern and traditional hives. Although it is important that beekeepers are trained in the appropriate bee husbandry skills for frame hives, otherwise the risk is that frame hives are managed much like traditional hives resulting in increased costs of hive purchase with no corresponding increase in yields. Previously beekeepers have incurred substantial losses due to the purchasing frame hives through loans without the requisite hive management skill-set. For example, beekeepers in the West Nile region of Uganda were offered loans to purchase modern hives, and subsequently failed to repay the debt, due to a lack of appropriate skills to manage such hives [29].

The provision of a year-round nectar source (*C*. *calothyrsus*) suggested that a non-negligible increase in revenue. For example addition of 2000 C. *calothyrsus* trees to the current state could potentially increase current revenues 7 times (Fig 7). An interesting observation is that beekeepers could generate more revenues if they combined addition of beehives, good pest and disease control and *Calliandra* trees to the baseline scenario. For example if a beekeeper added 2,000 trees + 20 log hives under good management they would increase their incomes 17.6 times much higher than just a single intervention. The role of bee forage in increasing honey yield is known [43,44]. Unknown to field extension workers in the regions was the effect of a density of trees i.e. how many bee forage plants with what combinations of hives does the beekeepers need to generate a specific amount of revenue? (Unpublished field observation Amulen). These models attempt to address the above question. However, there is need for field trials to validate the theoretical models.

When promoting the type of bee forage extension workers are advised to consider factors such as adoption, multi-purpose application as well land requirements to avoid compromising food production. The advantage of cultivating *C*. *calothyrsus* is that the plant is already used in the region as an incorporate of livestock feed [27,45], which would enhance its assimilation within farms [46], as farmers are more likely to adopt innovations they are familiar with [46]. *C*. *calothyrsus* is a multipurpose plant that can be used as a nitrogen fixer to improve soil fertility [47], and also a supply of firewood in a region where 90% of its cooking fuel is firewood [14] on top of providing bee forage [47]. Land is one of the key limitations of technology adoption e.g. adding trees on-farm [48]. Our proposed tree models (i.e. 500 tress, 1000 trees, 1500 trees and 2000 trees) require between 0.1 ha to 0.5 ha of land. We assumed beekeepers would utilize the land in between gardens (hedges) which is normally left uncultivated, reducing the potential of conflict between neighbours [48]. Adoption rates of *Calliandra* planting are likely to be restricted by several factors such as knowledge transfer of cultivation practices, seed supply, credit to purchase *Calliandra* tree seedlings as well as economic incentives to incite behaviour changes in traditional agricultural practices settings [49–53].

Whereas the modelled scenarios provide insights to the potential income streams, the current research challenge is to translate an idealized conception of production potential into practical reality. This will require an inter-disciplinary approach that embraces the scientific, political, developmental and public dimensions; hence the need for field testing of the scenarios to determine the exact effect of the interventions. Secondly, honeybees require diverse supplies of carbohydrates and proteins. In future models there is need to include diverse diets than one carbohydrate type to depict field situations, an aspect that was not possible for this current study due to limited literature on the reproductive and pollination biology of most bee forage plants under tropical conditions.

## Conclusion

This study evaluated the income generating potential of farmers through increased honey production in Northern Uganda. Increasing production volumes of hive products was contingent upon achieving the appropriate combination of hive type, number and the addition of a year-round forage crop *(C*. *calothyrsus)*. The study demonstrated that adoption of modern hive technologies should be driven by the beekeepers’ skill-level and financial capacity. For novice beekeepers, the use of traditional hives should be encouraged in order to minimize initial costs and to maximize the opportunity to acquire the appropriate expertise in bee handling. Top bar and frame hives can be introduced once beekeepers have developed their skill-set. Good management plus introduction of a good nectar source such as *Calliandra* could improve profitability of the beekeeping enterprise.

## Supporting information

S1 Questionnaire (DOCX)

S1 Table Household survey variables. (DOCX)
