# Supplementary material for: Estimating the potential of beekeeping to alleviate household poverty in rural Uganda
Source: PLoS One. 2019 Mar 27;14(3):e0214113. doi: 10.1371/journal.pone.0214113 (PMC6436742; doi:10.1371/journal.pone.0214113)
Supplement: S1 Questionnaire — (DOCX) [file pone.0214113.s001.docx]

## **S4: Household questionnaire**

The purpose of this questionnaire is to document the socio-economic characteristics of beekeeping and non-beekeeping households; perceptions, knowledge and attitudes of these households towards beekeeping; examine the current livelihood options available; barriers to women participation and their major sources of income.

**Introduction:**

Dear respondent this is to introduce *Ms Amulen Deborah Ruth* a graduate student of Ghent University conducting research on barriers to beekeeping in your region. The information obtained from this study will be handled with respect and confidentiality. It shall be used for academic purposes; with your consent, should I begin the interview? Yes ......... ….No……………..

**Questionnaire No._______________Locality________________GPS No:………………….**

**A. Socio-demographic characteristics of the respondents fill or tick in the adjacent boxes.**

| **Code** | **Attribute** |  | **Tick** |  | **Tick** |  | **Tick** |  | **Tick** |
| --- | --- | --- | --- | --- | --- | --- | --- | --- | --- |
| A.1 | Sex | **Female** |  | **Male** |  |  |  |  |  |
|  |  |  |  |  |  |  |  |  |  |
| A.2 | Age |  |  |  |  |  |  |  |  |
| A.3 | household head | Female |  | Male |  |  |  |  |  |
| A.4 | Marital Status | Single |  | Married |  | Divorced |  |  |  |
| A.5 | Household members |  |  |  |  |  |  |  |  |
| A.6 | Land ownership | Own land |  | Do not |  | Share land |  |  |  |
| A.7 | Land acreage |  |  |  |  |  |  |  |  |
| A.8 | Education level | No formal |  | Primary |  | Secondary |  | Tertiary |  |
| A.9 | Main income sources | On-farm |  | Off farm |  |  |  |  |  |
| A.10 | Years in beekeeping | **None** | <1year | 2-3years |  | 3-5years |  | >5years |  |

**B. Livelihood options, land allocation and economic contribution**

**B.1** Does this household engage in crop farming? **Yes = No =**

**B.2** If yes which crops are grown in this household? Tick in box below;

|  |  |  |
| --- | --- | --- |
|  | **Crop code** | **Tick crop grown** |
| 1 | Cassava |  |
| 2 | Sorghum |  |
| 3 | Millet |  |
| 4 | Sweet potatoes |  |
| 5 | Maize |  |
| 6 | Groundnuts |  |
| 7 | Beans |  |
| 8 | Cowpeas |  |
| 9 | Tobacco |  |
| 10 | Cotton |  |
| 11 | Simsim |  |
| 12 | Pigeon Peas |  |
| 13 | Other Crops |  |

**B.3.** Does this household keep livestock? **Yes = No =**

**B.4** If yes which livestock are reared in this household? Tick in the box below:

|  | **Livestock** | **Tick** | **Number of livestock** |
| --- | --- | --- | --- |
| 1 | Cattle |  |  |
| 2 | Sheep |  |  |
| 3 | Goats |  |  |
| 4 | Pigs |  |  |
| 5 | Poultry |  |  |
| 6 | Other |  |  |

**B.5** Does anyone in this household engage in off-farm activities? **Yes = No =**

**B.6** If yes, what are these non-farm activities? Tick and add them.

| **Code** | **Off farm employment** | **Tick** |
| --- | --- | --- |
| 1 | Small Business |  |
| 2 | Civil Servant |  |
| 3 | Charcoal Burning |  |
| 4 | Teaching |  |
| 5 | Politician |  |
| 6 | Brick Laying |  |
| 7 | Others |  |
|  |  |  |

**B.7.** What made you to choose the above crops and livestock? Tick and add list**.**

|  | **Reasons** | **Tick** |
| --- | --- | --- |
| 1 | Knowledge about It |  |
| 2 | Market available |  |
| 3 | Higher income |  |
| 4 | Household consumption needs |  |
| 5 | Culture |  |
| 6 | Interested |  |
| 7 | Status |  |

**B.8** Comparing crops and livestock; what uses most of your land? Fill the acres

| No | Enterprises | Acres |
| --- | --- | --- |
| 1 | Livestock |  |
| 2 | Crops |  |

**B.9** Where does money for this household come from? Fill table below;

| **No** |  | **Frequency of Income** | | | **Amount** |
| --- | --- | --- | --- | --- | --- |
|  | **Sources of Income** | **Monthly** | **Per season** | **Annually** |  |
| 1 | Crop sales |  |  |  |  |
| 2 | Livestock sales |  |  |  |  |
| 3 | Off farm employment |  |  |  |  |
| 4 | Other sources (non-farm employment) |  |  |  |  |

*fill in the frequency the farmer can remember

**Reasons for not adopting beekeeping: (non-beekeepers)**

**C.2** If you do not keep bees, what are your reasons? **If you keep bees go to C.4**

|  | **Attribute** | Tick |
| --- | --- | --- |
| 1 | Limited knowledge |  |
| 2 | No interest |  |
| 3 | Fear of bees |  |
| 4 | No capital |  |
| 5 | Limited space for beekeeping |  |
| 6 | No market for products |  |
| 7 | I Don’t think it can make money |  |
| 8 | Others |  |
|  | **Total** |  |

**Factors for attraction to beekeeping**

**C.3. For Non-Beekeepers:** Under what conditions would you consider starting beekeeping?

|  | **Conditions for beekeeping** | **Tick** |
| --- | --- | --- |
| 1 | Training on beekeeping |  |
| 2 | Market Availability |  |
| 3 | Land (Space) |  |
| 4 | Capital |  |
| 5 | Advisory support |  |
| 6 | Not Interested at all |  |
| 7 | Income from Bees |  |
| 8 | Time Availability |  |
| 9 | No Need I Am Rich |  |
| 10 | Security |  |
| 11 | **Others** |  |

**C.4 For Beekeepers:** If you keep bees, what attracted you to beekeeping? Tick and add if not on the list

|  | **Attribute** | Tick |
| --- | --- | --- |
| 1 | My parents |  |
| 2 | Training |  |
| 3 | Personal interest |  |
| 4 | Income |  |
| 5 | NGO’s |  |
| 6 | Others name them |  |
|  |  |  |

**Assessing social networks**

**C.7 Group membership:** **for beekeepers and non-beekeepers** are you a member of any of the following groups

|  | **Group** | Tick |
| --- | --- | --- |
| 1 | Farmers group |  |
| 2 | Marketing Group |  |
| 3 | Beekeepers association (for beekeepers) |  |
| 4 | Burial Group |  |
| 5 | Savings Group |  |

**Assessing the current knowledge level of beekeepers**

**C.8** Which aspects of beekeeping do you know? Please tick and add

|  | **Beekeeping knowledge** | **Tick** |
| --- | --- | --- |
| 1 | Local hive construction |  |
| 2 | Hive sitting |  |
| 3 | Capturing swarms |  |
| 4 | Pest and disease control |  |
| 5 | Honey harvesting and processing |  |
| 6 | Bee forage calendar |  |
| 7 | Other product processing |  |
| 8 | Proper hive inspection |  |
| 9 | Colony multiplication techniques |  |
| 10 | Feeding (water) |  |

**Assessing major sources of the current knowledge and skills**

**C.9** Where did you get this knowledge from? Please tick and add

|  | **Knowledge Source** | **Tick** |
| --- | --- | --- |
| 1 | Fellow beekeeper |  |
| 2 | From relative |  |
| 3 | Extension agent |  |
| 4 | Newspaper |  |
| 5 | Radios |  |
| 6 | Agricultural shows |  |
| 7 | Trial and error |  |

**Assessing Current Beekeeper Constraints**

**C.10** What problems do you face in beekeeping**? Choose at least 6**

|  | **Challenges** | **Tick** |
| --- | --- | --- |
| 1 | Aggressiveness of bees |  |
| 2 | Bush fires |  |
| 3 | Theft of hives and product |  |
| 4 | Drought |  |
| 5 | Limited knowledge |  |
| 6 | Pest and diseases |  |
| 7 | Limited space |  |
| 8 | Limited market for our products |  |

**Push factors for non-beekeepers not adopting beekeeping**

**C.11 For Non-beekeepers:** What are your fears of beekeeping? **Choose at least 6** add any (continue to **C 13**)

|  | **Challenge** | Tick |
| --- | --- | --- |
| 1 | Aggressiveness of bees |  |
| 2 | Bush fires |  |
| 3 | Theft of hives and products |  |
| 4 | I Have no knowledge |  |
| 5 | Not sure it is profitable |  |
| 6 | No space to place the beehives |  |
|  |  |  |

**Assessing beekeeper’s current investment capacity and sources of equipment**

**C.12** which of the following beekeeping equipment do you have?

| no | Materials | Tick | How many | home made | locally made& materials purchased | Provided on credit | donated | Cost | Number of years owned |  |
| --- | --- | --- | --- | --- | --- | --- | --- | --- | --- | --- |
| 1 | Log Hives |  |  |  |  |  |  |  |  |  |
| 2 | KTB Hives |  |  |  |  |  |  |  |  |  |
| 3 | Langstroth |  |  |  |  |  |  |  |  |  |
| 4 | Bee Veil |  |  |  |  |  |  |  |  |  |
| 5 | Gloves |  |  |  |  |  |  |  |  |  |
| 6 | Boots |  |  |  |  |  |  |  |  |  |
| 7 | Bee Overall |  |  |  |  |  |  |  |  |  |
| 8 | Water Sprayer |  |  |  |  |  |  |  |  |  |
| 9 | Airtight Bucket |  |  |  |  |  |  |  |  |  |
| 10 | Honey Strainer |  |  |  |  |  |  |  |  |  |
| 11 | Smoker |  |  |  |  |  |  |  |  |  |
| 12 | Bee Brush |  |  |  |  |  |  |  |  |  |
| 13 | Hive Tool |  |  |  |  |  |  |  |  |  |
| 14 | Honey Extractor |  |  |  |  |  |  |  |  |  |

**E. Extension service barriers**

**E.1:1** Do you have Access to any form of beekeeping extension services (Tick)

Yes

No

**E.2:** Which form of beekeeping extension services do you access? Tick and add if missing

|  | **Extension services** | **Tick** |
| --- | --- | --- |
| 1 | Training on management |  |
| 2 | Training on product processing |  |
| 3 | Routine visits by extension agent |  |
| 4 | Supply of beehives |  |
| 5 | Market information |  |
|  | Other |  |

**E.3:** Who provides these beekeeping extension services to you? Tick

|  | **Source of extension service** | Tick |
| --- | --- | --- |
| 1 | Government |  |
| 2 | NGOs |  |
| 3 | Private (community based) |  |
| 4 | Fellow Farmers |  |
| 5 | none |  |

**F. Bee products produced and marketing**

**F.1** Which Products do you harvest; what is the annual yield; what do you do to them? And what is the price per kg of each of the products?

|  |  |  | **Use** | |  |
| --- | --- | --- | --- | --- | --- |
|  | **Products** | **Quantity/ year** | **Home consumption** | **Sale** | **Price /kg** |
| 1 | Honey |  |  |  |  |
| 2 | Bees wax |  |  |  |  |
| 3 | Propolis |  |  |  |  |
| 4 | Pollen |  |  |  |  |
| 5 | Bees |  |  |  |  |

**F.4** Who buys your bee products? Tick

| **No** | **Buyer** | **Tick** |
| --- | --- | --- |
| 1 | Middlemen |  |
| 2 | Processing companies |  |
| 3 | Beekeepers cooperatives |  |
| 4 | Fellow members in community |  |
| 5 | Others specify |  |

**F.5** Which place do you sell your products from?

| **No** | **Places** | **Tick** |
| --- | --- | --- |
| 1 | At home |  |
| 2 | Nearby market |  |
| 3 | Agricultural shows |  |
| 4 | Village ceremonies |  |
| 5 | Others specify |  |

**F.6** What is the distance in kilometres from your home to the nearby market?

**………………………………………………………………………………………………………………………**

**F.7** How do you transport your products to the market?

| **No** | **Means of Transport** | **tick** |
| --- | --- | --- |
| 1 | Bicycle |  |
| 2 | Vehicle |  |
| 3 | Foot |  |
| 4 | Animal Traction |  |
| 5 | Others Specify |  |

**F.8** What constraints do you face in marketing your bee products? List them

| **No** | **Constraints** |  |
| --- | --- | --- |
| 1 | Market is far |  |
| 2 | Poor roads |  |
| 3 | Poor weather |  |
| 4 | Low demand |  |
| 5 | Product damages |  |
