# Supplementary material for: Estimating the potential of beekeeping to alleviate household poverty in rural Uganda
Source: PLoS One. 2019 Mar 27;14(3):e0214113. doi: 10.1371/journal.pone.0214113 (PMC6436742; doi:10.1371/journal.pone.0214113)
Supplement: S1 Table — (DOCX) [file pone.0214113.s002.docx]

S1 Table: Household survey variables

| **Variable** | **Response** |
| --- | --- |
| *Socio-demographic factors* |  |
| Age | (17-35years 1=yes, 0 =no), (36-55years 1=yes, 0 = no) (56 -70years 1=yes, 0 = no) & (>70years 1= yes, 0 = no) |
| Gender | (Female 1= yes, 0=no), (Male 1= yes, 0 =no) |
| Education | (No formal 1= yes 0 =no), (Primary 1=yes, 0=no), (secondary 1=yes, 0=no) & (Teartiary 1=yes, 0=no) |
| Number of beehives | numeric |
| Scale of beekeeping | (small scale beekeepers <22 beehives 1=yes, 0 =no), (large scale beekeepers > 22beehives 1 = yes, 0 =No) |
| Number of years in beekeeping (adopter categories) | (1-3 years late adopters 1=yes, 0=no), (4-7 years early adopters 1= yes, 0=no) (>8 years Innovators 1=yes, 0=no) |
| *Beekeepers knowledge* |  |
| Local hive contruction | (1= yes, 0=no) |
| Hive siting | (1= yes, 0=no) |
| Wild swarm capture | (1= yes, 0=no) |
| Pest control | (1= yes, 0=no) |
| Honey harvesting | (1= yes, 0=no) |
| Colony calendar | (1= yes, 0=no) |
| Product processing | (1= yes, 0=no) |
| Colony inspection | (1= yes, 0=no) |
| Colony multiplication | (1= yes, 0=no) |
| Colony feeding | (1= yes, 0=no) |
| *Beekeepers' group membership* |  |
| Farmers group | (1= yes, 0=no) |
| Marketing group | (1= yes, 0=no) |
| Beekeepers association | (1= yes, 0=no) |
| Savings and credit group | (1= yes, 0=no) |
| Burial group | (1= yes, 0=no) |
| *Number and unit cost of beekeeping equipment* |  |
| Beehives | numeric |
| log beehives | numeric |
| KTB hives | numeric |
| Langstroth hives | numeric |
| Pair of gumboots | numeric |
| Bee suits | numeric |
| Pair of gloves | numeric |
| Airtight bucket | numeric |
| Smoker | numeric |
| Bee brushes | numeric |
| Honey strainers | numeric |
| Hive tools | numeric |
| Honey extractors | numeric |
| Other costs |  |
| Bee colony maintance | numeric |
| labour (inspection and product harvest) | numeric |
| *Calliandra* seedling | numeric |
| Seedling planting labour | numeric |
| *Quantity of bee products and unit prices* |  |
| Honey | numeric |
| Beeswax | numeric |
| Propolis | numeric |
| Annual household incomes | numeric |
